# Retrospective analysis of non-tuberculous mycobacterial skin infections: diagnostic accuracy, treatment efficacy, and PCR-based species detection in a Chinese primary care hospital

**DOI:** 10.1128/jcm.01065-25

**Published:** 2025-11-13

**Authors:** Tianyi Xu, Yingjie Zheng, Ruoning Xue, Ruoyu Li, Wen Zhang, Zaihong Sun, Yinggai Song

**Affiliations:** 1 Department of Dermatology and Venerology, Peking University First Hospital 26447, Beijing, China; 2 Research Center for Medical Mycology, Peking University 12465, Beijing, China; 3 National Clinical Research Center for Skin and Immune Diseases, Beijing, China; 4 Dermatosis Prevention and Control Station of Shouguang, Weifang, Shandong Province, China; University of Western Australia, Perth, Australia

**Keywords:** primary care, empirical treatment, PCR-based diagnosis, skin infections, Non-tuberculous mycobacteria (NTM)

## Abstract

Non-tuberculous mycobacterial (NTM) skin infections present diagnostic and therapeutic challenges in resource-limited primary care settings. This retrospective study evaluated the diagnosis and treatment of NTM skin infections at a primary care hospital in Shouguang City, a coastal region of China with high-risk occupational exposure to aquatic environments, from 2021 to 2022. We analyzed 144 patients, using real-time PCR on formalin-fixed paraffin-embedded (FFPE) tissue samples to validate empirical diagnoses based on clinical symptoms, exposure history, and histopathology. Our results demonstrated a high concordance (79.17%) between empirical and molecular diagnoses, confirming the reliability of the primary care hospital’s diagnostic approach. Real-time PCR successfully identified specific NTM species in a subset of cases, with *Mycobacterium marinum* and *Mycobacteroides abscessus* being the most prevalent. Empirical treatment with combination antibiotics (primarily clarithromycin and rifampicin) was highly effective, achieving a cure rate of 90.97%. This study highlights the effectiveness of integrated clinical-histopathological assessment for managing NTM infections in resource-limited settings and underscores the value of molecular techniques such as PCR in enhancing diagnostic precision and facilitating retrospective research using archived FFPE samples.

**IMPORTANCE:**

This study addresses a critical gap in managing non-tuberculous mycobacterial (NTM) skin infections in resource-limited primary care settings, with particular relevance to coastal regions like Shouguang, where occupational exposure to marine environments elevates infection risk. By validating empirical diagnosis through molecular testing, we demonstrate that integrated clinical-histopathological assessment is a reliable method for identifying NTM infections where advanced diagnostics are unavailable. Our adaptation of real-time PCR to archived FFPE tissues provides a feasible model for retrospective species identification and research, overcoming key resource constraints. The high documented cure rate with clarithromycin-rifampicin regimens provides practical treatment benchmarks for regions with similar epidemiological profiles. These findings offer immediate clinical value by: (i) reinforcing the reliability of empirical diagnosis in resource-limited contexts, (ii) establishing a practical pathway for molecular confirmation using stored samples, and (iii) validating effective treatment protocols for high-risk occupational groups. As NTM incidence increases globally, this work equips primary care systems with evidence-based strategies to improve diagnostic accuracy and patient outcomes in settings with high marine exposure while simultaneously enabling future research through the utilization of existing biospecimen repositories.

## INTRODUCTION

Non-tuberculous mycobacteria (NTM) are mycobacteria other than *Mycobacterium tuberculosis* and *Mycobacterium leprae*, widely found in natural environments (1–3). As opportunistic pathogens, NTM can cause diverse diseases, including pulmonary, cutaneous (4), and disseminated infections, posing diagnostic and treatment complexities (5). With advancements in medical technology and an increase in susceptible populations, NTM infection rates have increased annually, presenting significant public health challenges (6–8).

In China, pulmonary infections, particularly non-tuberculous mycobacterial pulmonary disease (NTM-PD), are the most common NTM infections, with skin infections also prevalent (9, 10). The most frequently isolated NTM species include *Mycobacterium intracellulare*, *Mycobacteroides abscessus,* and *Mycobacterium kansasii*, with regional variations. *Mycobacteroides chelonae/abscessus* is more common in the south, whereas *M. kansasii* has a higher isolation rate in the north (11).

China’s primary hospitals face challenges in NTM diagnosis and treatment due to limited resources, insufficient testing equipment, and specialized personnel, particularly in coastal cities. They often rely on traditional smear and culture methods, which are limited in both efficiency and accuracy. Despite these challenges, primary hospitals play a crucial role in initial patient visits, screening, and referrals, providing timely interventions and reducing the burden on higher-level hospitals.

This study retrospectively investigated the diagnosis and treatment of NTM skin infections at a primary care hospital in Shouguang City—a coastal region of Shandong Province, China, with high prevalence of NTM infections associated with aquatic occupational exposures—from 2021 to 2022. We re-sliced stored formalin-fixed paraffin-embedded (FFPE) skin tissue samples, extracted DNA, and used NTM-specific primers designed by our research group to apply real-time PCR technology (12). This allowed us to detect the presence of NTM and specific species in the samples. The aim was to assess the effectiveness of empirical diagnosis and treatment based on clinical symptoms and pathology slides in a resource-limited setting. Notably, this study innovatively used non-fresh, well-preserved FFPE samples for PCR species detection. This approach extends the application of our previously established PCR assay from fresh skin tissue samples to retrospective studies and provides an efficient and reliable method for improving the diagnostic accuracy of NTM infections.

## MATERIALS AND METHODS

### Case screening and data analysis

In this study, a total of 144 patients with NTM infections who attended Shouguang Dermatology Hospital in Shandong Province from January 2021 to December 2022 were selected. The case inclusion criteria were as follows: (i) with suspected clinical manifestations of skin infection; (ii) histopathologic examination showed infected granulomas, including tuberculous granuloma or *tuberculosis*-like granuloma; and (iii) the patients responded well to antimycobacterial drug therapy during the follow-up period.

Patient data were collected through the outpatient medical record system and included basic information such as gender, age, date of visit, exposure history, site of onset, type and number of lesions, and treatment. Clinical photographs were kept for each patient. Patients were managed through regular follow-up visits or telephone calls and continued to be followed up for 6 months after healing. Prior to the implementation of real-time PCR testing, patient information was supplemented by telephone follow-up, and recurrence was verified. By the time of real-time PCR testing, all patients had been followed up for more than 6 months, with the longest follow-up period being two and a half years.

The criteria for determining the efficacy of the treatment are as follows: (i) cured: after anti-NTM treatment, the rash subsides completely (pigmentation or scarring may remain), and (ii) effective: after treatment, the extent of the rash is significantly reduced compared with the pre-treatment area, or the rash flattens out, or the swelling is significantly reduced. Some patients who stopped the drug during the treatment when the rash improved but did not completely subside, and then the rash worsened, were still judged to be effective. All cured and effective patients were diagnosed with NTM infection if they met both the clinical manifestations and histopathologic features of NTM infection.

### DNA extraction of tissue samples

In this study, skin tissue wax blocks from 144 patients who met the screening criteria were obtained from the repository. These wax blocks were re-sliced, and the thickness of the slices was controlled at 6–8 µm, and 10 slices were cut for each sample. DNA extraction of formalin-fixed and paraffin-embedded (FFPE) skin tissue sections was performed using the DNA FFPE Tissue Kit (Qiagen, 56404, USA). The extraction process was strictly followed, according to the instructions provided with the kit. The extracted DNA samples were dissolved in ultrapure water in a final volume of 100 µL per sample. Subsequently, the DNA samples were quantified, and their purity was determined using a Nanodrop spectrophotometer.

### Fluorescence real-time PCR

In this study, fluorescent real-time PCR was performed on the DNA of skin tissue samples from 144 enrolled cases. In the PCR reaction system, the following components were included: 2 µL of 15-fold diluted tissue DNA samples, 0.4 µL each of forward and reverse primers (both 10 µmol/L), and 0.4 µL of probe (10 µmol/L), 10 µL of 2× SuperMix (TransGen Biotech, China), Passive Reference Dye II 0.4 µL, and the remaining volume was made up to 20 µL with ultrapure water. The primers and probes used in this study were designed by our research group and have been previously described in a study by Liu Xiao et al. (12) and are currently protected under a Chinese patent (Patent holders: Yinggai Song, Xiao Liu, Ruoyu Li. Title: Primers and probes for pathogenic bacteria in infectious granuloma of skin, implementation method, and detection system. Patent No. ZL202110452654.8 [P]. 2022-08-30). Due to patent protection, the primer sequences cannot be provided in this publication. Although the exact nucleotide sequences of the primers and probes are protected under a patent and cannot be disclosed, the specific genetic targets and the mycobacterial species detected by each set are detailed in Table 1 to ensure scientific transparency and reproducibility.

**TABLE 1:** Genetic targets of the primers and probes used for *Mycobacterium* species identification

Primers	Species	Target gene
16 s	*Mycobacterium spp*.	16S rRNA
TB	*M. tuberculosis*	Hypothetical protein gene
Mar	*M. marinum*	Type I polyketide synthase
Ulc	*M. ulcerans*	Type I polyketide synthase
Avi	*M. avium*	DNA-directed RNA polymerase subunit beta
Hem	*M. haemophilum*	Membrane protein gene
Kan	*M. kansasii*	Before 23S rRNA
Int	*M. intercelleulare*	Homocysteine methyltransferase gene
Che	*M. chelonae*	Polymerase III subunit beta gene
For	*M. fortuitum*	Before hypothetic protein gene
Mal	*M. malmoense*	Methyltransferase gene
Szu	*M. szulgai*	Before 16S rRNA
Sme	*M. smegmatis*	Before methyltransferase gene
Gor	*M. gordonae*	Before 23S rRNA
Abs	*M. abscessus*	After 16S rRNA

The amplification process was performed using an ABI Vii7 fluorescence real-time quantitative PCR instrument (ABI, USA). The RT-PCR reaction conditions were set as follows: first, a pre-denaturation at 95°C was performed for 10 min, and this step was performed for one cycle; then, a 40-cycle amplification step was carried out, and each cycle consisted of denaturation at 95°C for 15 s, and annealing and extension at 60°C for 1 min.

## RESULTS

### Patient characteristics and epidemiological analysis

A total of 144 patients with clinically and histopathologically diagnosed NTM skin infections were included in this retrospective study. The baseline demographic and epidemiological characteristics of the patient cohort are summarized in Table 2. Briefly, there was a slight female predominance (59.03%). The majority of patients were farmers (61.81%), and a clear history of preceding trauma was documented in 61.81% of cases. Notably, over half of these traumatic events (53.93% of those with trauma history) were directly associated with handling aquatic products.

**TABLE 2:** Baseline characteristics and epidemiological information of patients

Category	Details	Number (n)	Percentage (%)
Total patients		144	100
Gender	Male	59	40.97
	Female	85	59.03
Age (years)	Mean	61.5	
	Range	32-81	
Occupation	Farmers	89	61.81
	Supermarket employees	15	10.42
	Nonaquatic industry workers	15	10.42
	Chefs	5	3.47
	Construction workers	5	3.47
Trauma history	Present	89	61.81
	– Aquatic product-related		48 (53.93^*a*^)
	– Other trauma with aquatic exposure		3 (3.37^*a*^)
	– Other forms of trauma		37 (41.57^*a*^)
	Absent	55	38.19

^*a*^ Note: Percentages for trauma subtypes are calculated against the total number of patients with a trauma history (*n* = 89).

### Clinical manifestations and pathological features

The clinical characteristics of the 144 patients are summarized in Table 3. The infections predominantly presented as infiltrative plaques and nodules, with a minority of cases exhibiting mild ulceration. A significant majority of lesions (88.89%) were unilateral, with the hand and wrist area being the most commonly involved site (70.83%), a finding consistent with the high prevalence of aquatic product-related trauma in this cohort. The fixed type was the most common clinical pattern (60.42%).

**TABLE 3:** Clinical characteristics of patients with NTM skin infections (*n* = 144)

Characteristic	Category	Number of cases	Percentage (%)
Lesion type	Infiltrative plaques/Nodules	125	86.81
	Ulcers	19	13.19
Distribution	Unilateral	128	88.89
	Bilateral	15	10.42
	Mid-facial	1	0.69
Site of involvement	Hand to wrist	102	70.83
	Upper limbs only	10	6.94
	Hand-Upper limbs	26	18.06
	Face	3	2.08
	Lower limbs	2	1.39
	Extremities	1	0.69
Clinical pattern	Fixed type	87	60.42
	Lymphatic spread type	56	38.89
	Disseminated type	1	0.69

**Fig 1:**
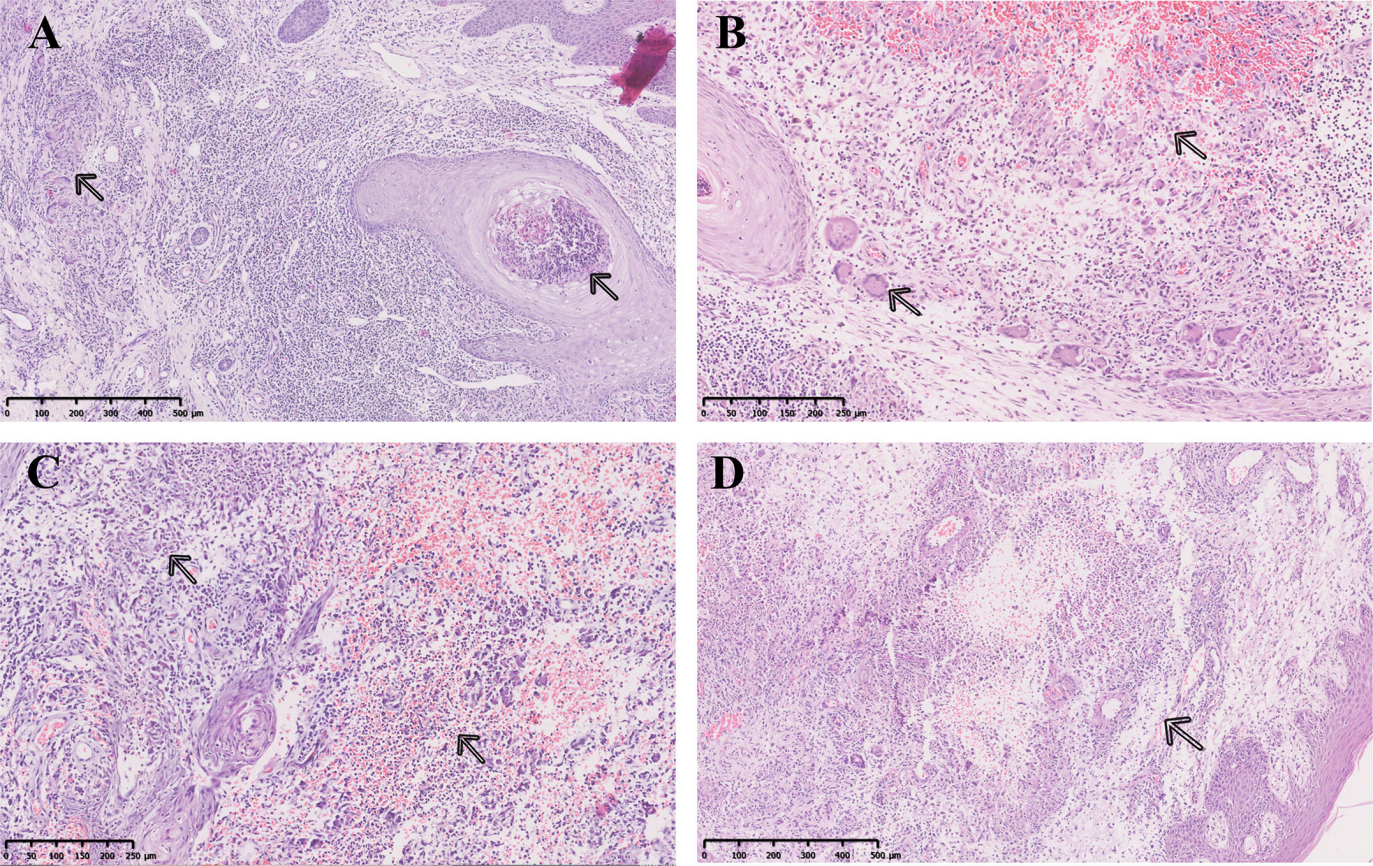
Typical pathological manifestations of NTM skin infections. (**A**) Epidermal hyperplasia with neutrophilic microabscesses; dermal histiocytic granulomas and multinucleated giant cells with lymphocytic infiltrate. (**B**) Large dermal histiocytic granuloma containing scattered multinucleated giant cells and neutrophils. (**C**) Multiple dermal neutrophilic microabscesses and histiocytic granulomas with perimicroabscess histiocytic infiltration. (**D**) Multiple dermal histiocytic granulomas with peripheral lymphocytic infiltrate.

Histopathological examination revealed that all cases showed features consistent with infected granulomas. These were characterized by irregular epidermal hyperplasia and granulomatous infiltration in the dermis, composed of large numbers of lymphocytes, histiocytes, and multinucleated giant cells. Additional findings observed in some cases included caseous necrosis-like necrosis, with central necrotic foci surrounded by epithelioid cells and multinucleated giant cells, as well as a few neutrophilic microabscesses (Fig. 1). However, due to the limitations of acid-fast stain, definitive species identification was not possible at the histopathologic level. Therefore, the empirical diagnosis of NTM infection was made by integrating these characteristic histopathologic findings with the patient’s clinical presentation and a history of potential exposure.

### Diagnostic, therapeutic practices, and outcomes

At this primary care hospital in Shandong Province, the diagnosis of NTM infections was primarily based on clinical symptoms, exposure history, and histopathological examination. Physicians made empirical diagnoses by integrating patients' clinical manifestations, such as the presence of skin lesions (plaques, nodules), exposure to potential sources of NTM (e.g., fish, shrimp, and wood splinters), and histopathological findings indicative of granulomatous inflammation. For instance, in patients with a history of aquatic product injuries, the presence of typical skin lesions and granulomas on histopathology supported the empirical diagnosis of NTM infection.

The treatment regimens at this primary care hospital were mainly empirical, targeting the suspected NTM pathogens. The most commonly used antibiotics included clarithromycin and rifampicin, often in combination. For example, a typical treatment regimen might consist of clarithromycin 500 mg twice daily and rifampicin 450 mg twice daily. A small proportion of patients (less than 10%) also received adjunct antibiotics such as minocycline or moxifloxacin, depending on the clinical judgment and response to initial treatment. The duration of treatment varied, with most courses lasting from 1 to 10 months.

The therapeutic outcomes were generally favorable, with a high rate of clinical improvement. Out of the 144 patients, 131 (90.97%) achieved clinical cure, defined as complete resolution of skin lesions with no new lesions appearing during follow-up. Thirteen patients (9.03%) showed improvement but did not achieve a complete cure; some of these patients had partial resolution of lesions but experienced recurrence due to irregular medication intake or self-discontinuation of treatment. Notably, five patients who had stopped taking medication midway experienced a relapse of skin lesions, which resolved after resuming treatment and did not recur during the subsequent 6-month follow-up period.

### PCR validation and diagnostic accuracy

To molecularly validate the empirical diagnoses, we performed real-time PCR on DNA extracted from all 144 FFPE tissue samples. The assay confirmed the presence of NTM DNA in 114 cases (79.17%), demonstrating high concordance between clinical and molecular diagnoses. Among the PCR-positive cases, 30 (26.32%) were successfully identified to the species level (Table 4). The majority of speciated cases were *Mycobacterium marinum* and *M. abscessus*, whereas the remaining PCR-positive cases (73.68%) were identified only to the genus level. This limitation may be attributed to low bacterial DNA load, non-targeted or novel species not covered by our primer-probe panel, DNA degradation in FFPE tissues, or genetic similarities between mycobacterial species. Nonetheless, the ability to achieve species-level identification in over a quarter of archived FFPE samples highlights the utility of this molecular approach for retrospective studies. Future investigations could employ sequencing of targets such as the 16S–23S rRNA ITS region or rpoB gene for precise species classification of these samples.

**TABLE 4:** Real-time PCR detection results

Category	Details	Number (n)	Percentage (%)
PCR results	NTM-positive (16S rRNA)	114	79.17
	NTM-negative	30	20.83
Species identification	Identified to genus level only	84	73.68^*a*^
	Identified to species level	30	26.32^*a*^
Species distribution	*M. marinum*	16	
	*M. abscessus*	4	
	*M. tuberculosis*	2	
	*M. gordonae*	2	
	*M. kansasii*	2	
	*M. smegmatis*	1	
	*M. malmoense*	1	
	*M. chelonae*	1	
	*M. szulgai*	1	

^*a*^ Note: Percentages for species identification are calculated against the total number of PCR-positive cases (*n* = 114).

**Fig 2:**
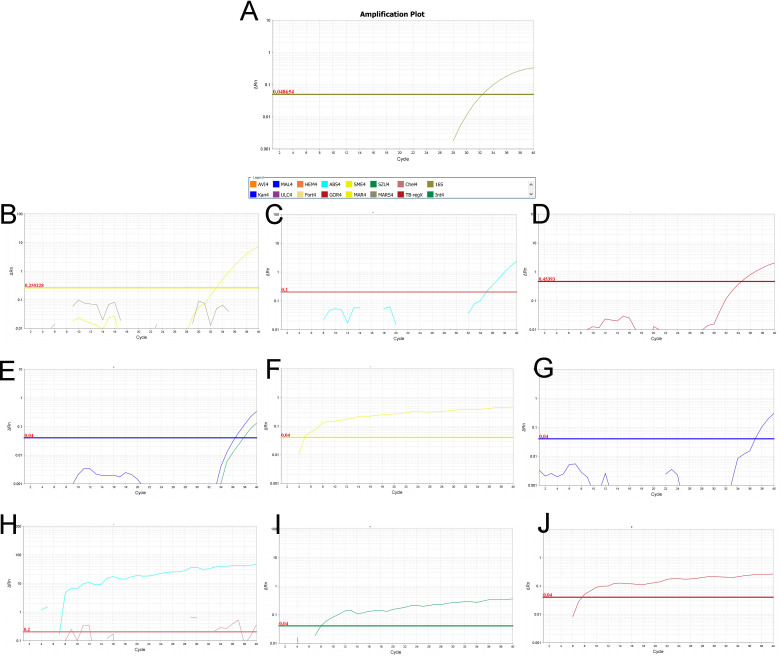
PCR amplification plot for NTM detection. (**A–J**): PCR amplification plots for the 16S rRNA gene and specific NTM species (MAR, ABS, GOR, KAN, SME, MAL, CHE, SZU, and TB). Each plot represents the detection of the corresponding NTM species or the genus level.

This molecular validation underscores the reliability of the diagnostic approach integrating clinical symptoms, exposure history, and histopathology. Representative amplification plots for the *Mycobacterium* genus (16S rRNA) and specific NTM species are shown in Fig. 2, illustrating the robustness and specificity of the method.

## DISCUSSION

The diagnosis and management of NTM skin infections pose considerable challenges in primary care settings, particularly where laboratory resources are limited. This retrospective study, conducted in a primary care hospital in China, demonstrated a 79.17% concordance rate between clinical diagnosis—based on exposure history, histopathology, and treatment response—and PCR confirmation. The selection of the empirical clarithromycin/rifampicin regimen was based on the 2024 Chinese expert consensus on the diagnosis and treatment of cutaneous NTM diseases, which specifically recommends this combination as the first-line therapy for the most prevalent species in coastal regions of China, particularly *M. marinum* (13). This regimen achieved a 90.97% cure rate, affirming the practicality of empirical approaches in resource-constrained environments (14–17). The consensus further supports this approach in primary care settings where species identification capabilities are limited while acknowledging the importance of susceptibility testing when available, especially for *M. abscessus* complex, which may harbor inducible macrolide resistance mechanisms. The species distribution, dominated by *M. marinum* and *M. abscessus*, aligns with established epidemiological patterns linking NTM skin infections to aquatic exposure or traumatic injury (18–22) while also reflecting regional ecological characteristics.

The diagnostic performance of various real-time PCR methods shows considerable variation, with accuracy highly dependent on target gene selection, primer design, and assay optimization (23, 24). Multiple studies have demonstrated that some commercial kits, such as GENEDIA MTB/NTM, exhibit limited sensitivity for NTM detection in sputum samples (25), whereas others, like VIASURE MTBC + NTM, perform well with culture-positive specimens (26), yet remain challenging to apply directly to FFPE tissue samples—the most accessible biological specimens in primary care settings. Notably, newly developed multi-target PCR systems (simultaneously targeting MTB complex-specific IS6110 and NTM-conserved rpoB genes) and multiplex LAMP technology have shown significant advantages in improving species differentiation accuracy (11, 27). However, most of these methods still require fresh samples or complex procedures, remaining limited in retrospective diagnosis using routine pathological archive materials.

In contrast, our study innovatively applied an optimized real-time PCR method to FFPE tissue sections—the most readily available biological sample type in primary hospitals—achieving successful NTM detection in 79.17% of cases and species identification in 26.32% of positive samples. This strategy not only overcomes the dependency of conventional PCR on fresh samples but also significantly enhances the accessibility and practicality of molecular diagnosis in resource-limited settings while maintaining high detection consistency.

Traditional detection methods, such as acid-fast bacilli smear and NTM culture, are limited by their poor specificity, long detection time (28), and inability to accurately distinguish between MTB and NTM or identify mixed infections (29). Although emerging technologies like MALDI-TOF MS (4- to 6-h turnaround time) (30), Sanger sequencing (31), and whole-genome sequencing (WGS) (costing $200–$500 per sample) (32) offer improved performance, their implementation remains limited by equipment requirements and operational costs. Beyond PCR, alternative molecular methods such as loop-mediated isothermal amplification (LAMP) have shown promise in resource-limited settings due to their competitive sensitivity and lower cost (33). Additionally, interferon-gamma release assays have demonstrated utility in specific scenarios for distinguishing NTM infections (34).

It is important to emphasize that molecular methods should complement rather than replace traditional culture. As demonstrated by the study from Hangzhou (35), medical personnel should not overestimate the value of metagenomic next-generation sequencing (mNGS) in small patient populations and should pay more attention to routine culture methods, particularly in the selection of appropriate temperature and medium types. Culture remains essential for obtaining isolates for drug susceptibility testing, which is critical for guiding therapy, particularly in drug-resistant or atypical cases. This is consistent with the 2024 Chinese consensus, which emphasizes that although empirical therapy with clarithromycin/rifampicin is appropriate for typical presentations in endemic areas, culture and susceptibility testing should be pursued when there is poor treatment response or clinical deterioration. Although the real-time PCR approach used in this study offers advantages in rapid diagnosis and species identification, its implementation in primary care is hindered by challenges such as DNA degradation in FFPE samples, high costs, and dependency on specialized equipment. Future efforts should focus on optimizing DNA extraction protocols, streamlining procedures, and reducing expenses to improve feasibility.

This study establishes a practical diagnostic framework that combines molecular validation of FFPE samples with clinical assessment, creating a viable model for primary care that balances accuracy and operational feasibility. Our findings, supported by the 2024 Chinese consensus, demonstrate that in resource-limited settings with high prevalence of aquatic-related NTM infections, an integrated approach combining empirical therapy based on consensus guidelines with available diagnostic technologies can achieve favorable patient outcomes. The molecular diagnosis of NTM infections benefits from a diversified suite of solutions: high-throughput technologies for central laboratories, standardized algorithms for reference facilities, and FFPE-compatible methods for grassroots settings. Continued optimization of context-appropriate diagnostic strategies will be essential to advancing global equity in the management of NTM infections.

## SUPPLEMENTAL MATERIAL

Table S1 jcm.01065-25-s0001.xlsx Basic information, clinical characteristics, and treatment outcomes of NTM skin infection cases.

ASM does not own the copyrights to Supplemental Material that may be linked to, or accessed through, an article. The authors have granted ASM a non-exclusive, world-wide license to publish the Supplemental Material files. Please contact the corresponding author directly for reuse.
